# Thoracic Computed Tomography to Assess ARDS and COVID-19 Lungs

**DOI:** 10.3389/fphys.2022.829534

**Published:** 2022-05-02

**Authors:** Carmen Silvia Valente Barbas

**Affiliations:** Associate Professor of Pneumology, University of São Paulo Medical School, Medical Staff Adult ICU Albert Einstein Hospital, São Paulo, Brazil

**Keywords:** ARDS, COVID-19, thoracic-computed tomography, pulmonary dual-energy angio-tomography, mechanical ventilation, patient position, PEEP, recruitment

## Abstract

This review was designed to discuss the role of thoracic-computed tomography (CT) in the evaluation and treatment of patients with ARDS and COVID-19 lung disease. Non-aerated lungs characterize the ARDS lungs, compared to normal lungs in the lowermost lung regions, compressive atelectasis. Heterogenous ARDS lungs have a tomographic vertical gradient characterized by progressively more aerated lung tissues from the gravity-dependent to gravity-independent lungs levels. The application of positive pressure ventilation to these heterogeneous ARDS lungs provides some areas of high shear stress, others of tidal hyperdistension or tidal recruitment that increases the chances of appearance and perpetuation of ventilator-induced lung injury. Other than helping to the correct diagnosis of ARDS, thoracic-computed tomography can help to the adjustments of PEEP, ideal tidal volume, and a better choice of patient position during invasive mechanical ventilation. Thoracic tomography can also help detect possible intra-thoracic complications and in the follow-up of the ARDS patients’ evolution during their hospital stay. In COVID-19 patients, thoracic-computed tomography was the most sensitive imaging technique for diagnosing pulmonary involvement. The most common finding is diffuse pulmonary infiltrates, ranging from ground-glass opacities to parenchymal consolidations, especially in the lower portions of the lungs’ periphery. Tomographic lung volume loss was associated with an increased risk for oxygenation support and patient intubation and the use of invasive mechanical ventilation. Pulmonary dual-energy angio-tomography in COVID-19 patients showed a significant number of pulmonary ischemic areas even in the absence of visible pulmonary arterial thrombosis, which may reflect micro-thrombosis associated with COVID-19 pneumonia. A greater thoracic tomography severity score in ARDS was independently related to poor outcomes.

## Introduction

**FIGURE 1:**
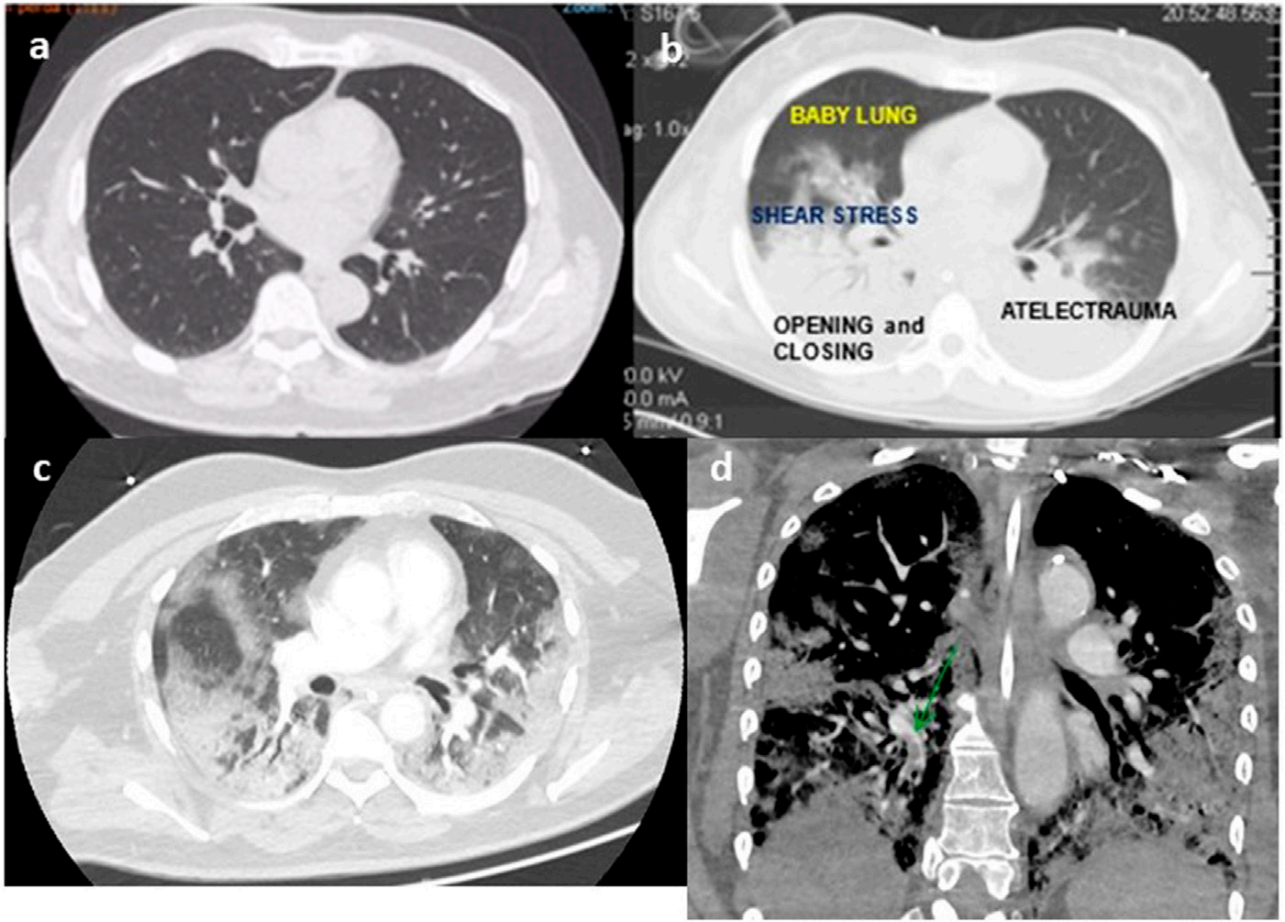
Thoracic tomography of a normal person **(A)**, person with ARDS **(B)**, person with COVID-19 **(C)**, and angio-tomography with pulmonary embolism **(D)** (authors own material from reference 14,17 and 45).

Since 2012, the diagnosis of acute respiratory distress syndrome (ARDS) is made by the finding of a recent (less than 1 week) bilateral lung infiltrates in the chest radiography of a patient with PaO2/FIO2 less than 300 and a risk factor for ARDS according to Berlin definition (Ranieri et al., 2012). This new re-classification of ARDS (Ranieri et al., 2012) recognized that bilateral opacities consistent with pulmonary edema on the chest radiograph as defining criteria for ARDS could also be demonstrated on thoracic-computed tomography. The images of thoracic-computed tomography in patients with ARDS helped to confirm the diagnosis of ARDS (Bombino et al., 1991; Barbas et al., 2003; Caser et al., 2014; Barbas et al., 2014; Pesenti et al., 2016) (Figure 1), detecting parenchymal and interstitial alterations as well as the quantification of the amount and regional lung distribution of the non-aerated, poorly-aerated, aerated, and hyperinflated lung tissue (Pesenti et al., 2016). Moreover, thoracic-computed tomography can help to differentiate ARDS from cardiogenic pulmonary edema, alveolar hemorrhage, and acute interstitial pneumonia (Barbas et al., 2003).

In 2003, Pelosi et al. (2003) reviewed pulmonary (caused by pneumonia, aspiration) and extra-pulmonary (caused by sepsis, pancreatitis) ARDS, they reported that a prevalent alveolar consolidation characterized the radiological presentation of pulmonary ARDS while the extra-pulmonary ARDS was characterized by ground-glass opacities. They also reported that in pulmonary ARDS the lung compliance was decreased, while in extra-pulmonary ARDS the chest wall and intra-abdominal chest compliance were decreased. They also showed that the effects of positive end-expiratory pressure (PEEP), recruitment maneuvers, and prone position were greater in extra-pulmonary ARDS (Pelosi et al., 1994).

So, this mini-review aims to discuss crucial articles that helped to better understand the role of thoracic-computed tomography to improve the diagnosis of the syndrome ARDS and COVID-19 lungs, to better understand its pathophysiology and the distribution of pulmonary edema according to the gravity force in different positions of the patients in the ICU bed and the ARDS lungs heterogeneity that could induce and perpetuate ventilator lung injury during inadequate positive pressure ventilation. This mini-review also discusses the role of thoracic tomography in detecting complications, prognosticating ARDS, and the part of dual-energy angio-tomography in COVID-19 patients.

## Quantitative Thoracic-Computed Tomography Analysis in ARDS

A pathophysiological hallmark of ARDS is the increased permeability of the alveolo-capillary membrane, leading to interstitial and alveolar flooding with edema rich in proteins and consequent collapse of the bottom areas of the lungs (Pesenti et al., 2016), shunt, and hypoxemia. In studying thoracic tomography of ARDS patients, the Gattinoni’ s group showed a greater vertical gradient of regional lung inflation in ARDS patients than normal patients (Cressoni et al., 2014). ARDS lungs compared to normal lungs are characterized by non-aerated lungs in the lowermost lung regions, compressive atelectasis, and progressively more aerated lung tissues from the lower to upper lung levels characterizing the ARDS heterogenous lungs (Cressoni et al., 2014), with areas of possible high shear stress and possible ventilator-induced lung injury during positive pressure ventilation.

The Gattinoni’s group reported the effects of PEEP on regional distribution of tidal volume and recruitment, while increasing PEEP from 0 to 20 cm H20, they observed that tidal volume distribution decreased significantly in the upper lungs regions, did not change in the middle levels, and increased significantly in the lower lungs regions. Studying ARDS patient’s lung recruitability and CT scan-derived PEEP, they observed that to overcome ARDS lungs compressive forces and to lift up the thoracic cage, a similar amount of PEEP was required in higher and lower recruiters (16.8 ± 4 vs. 16.6 ± 5.6 cm H20, *p* = 1) (Gattinoni et al., 1995). The Gattinoni’s group also reported the analysis of 68 patients who underwent whole-lung CT during breathing–holding sessions at airway pressures of 5, 15, and 45 cm H20, showing that the lung recruitability is heterogeneous and associated with the response to PEEP (Gattinoni et al., 2006).

**FIGURE 2:**
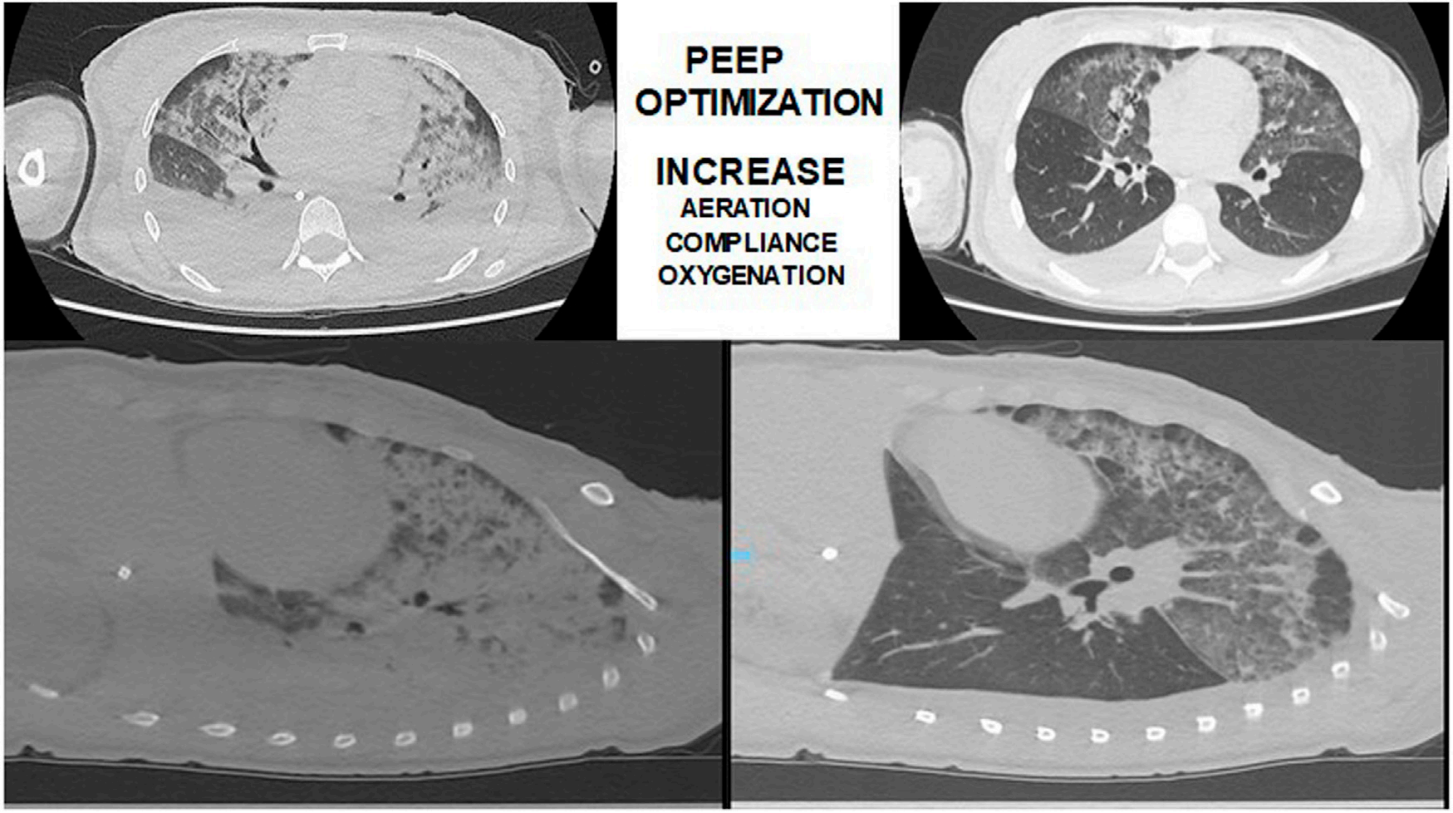
Thoracic-computed tomography guided recruitment and PEEP titration in a patient with ARDS (authors own material from reference 14).

Studies from Borges et al. (2006) and de Matos et al. (2012) reported that most of the collapsed lung tissue observed in early ARDS could be reversed using an image-monitored recruitment maneuver. After the lungs were opened up, a decremental PEEP titration maneuver guided by thoracic tomography was made to guarantee the lungs open, with less than 5% of collapsed lungs tissue. De Matos reported a case series of 51 ARDS patients, in which PEEP titration was guided by CT scan suggesting that it could be a good option to adjust PEEP and positive pressure ventilation in difficult and complex cases of ARDS (de Matos et al., 2012) (Figure 2). The ART, prospective, controlled, and clinical trial in ARDS patients (Cavalcanti et al., 2017) reported that unmonitored recruitment maneuvers with PEEP titration to the best compliance (titrated by respiratory mechanics and not guided by thoracic tomography) resulted in increased mortality compared to the control group. Therefore, it is not recommended carrying out lung recruitment maneuvers in invasively ventilated ARDS patients without respiratory image monitoring in ARDS patients.

By studying thoracic tomography of ARDS patients in the prone position (Langer et al., 1988; Gattinoni et al., 2013a; Gattinoni et al., 2013b), it has been shown that in the prone position, computed thoracic tomography scan densities redistribute from the dorsal to ventral regions of the lungs as the dorsal region tends to reexpand preventing the ventral zone to hyperinflate. Recently, Guérin et al. (2013) studied early ARDS patients with PaO2/FIO2 less than 150, with PEEP > than 5 cm H20 comparing mechanical ventilation in the prone position (periods of 16 h) vs. the supine position showed a significant improvement in survival rates in the patients submitted to the prone position.

The quantitative thoracic-computed tomography in ARDS patients improved the knowledge of pathophysiology of ARDS, showing a vertical gradient from the most gravity-dependent parts of the lung to the independent parts of the lungs, and the studies of different tidal volumes, PEEPs, recruitment maneuvers, and the influence of the prone position helped to guide clinical studies testing different approaches of invasive mechanical ventilation in the evolution and prognosis of ARDS patients (Amato et al., 1998; Barbas et al., 2012; Barbas and Nemer, 2018; Barbas et al., 2019; Pelosi et al., 2021).

## Thoracic-Computed Tomography to Detect Complications of ARDS and Possible Complications Related to Radiation Exposure

Simon et al. (2016), analyzing 204 thoracic-computed tomography of ARDS patients that full filled the Berlin definition criteria reported that the most common alterations of the lungs parenchyma were consolidations (94.1% of cases) and ground-glass opacities (85.3%). They also observed pleural effusions, mediastinal lymphadenopathy, signs of right ventricular strain and pulmonary hypertension, pericardial effusion, emphysema of the chest wall, pneumothorax, emphysema of the mediastinum, and pulmonary embolism, resulting in the change of ARDS patients’ management about 26.5% of cases. Chest CT allows the localization of invasive chest devices. Patients with more than 80% of involvement of the lung parenchyma had a significant increase in mortality (*p* < 0.004. Intrahospital transport was associated with critical incidents in 8.3% of cases (Simon et al., 2016).

Regarding the risks of radiation exposure, Chiumello et al. (2014) showed that a 70% reduction in the effective dose of radiation can be achieved in patients with ARDS during the acquisition of low-dose chest CT images, maintaining lung quantitative and anatomical results. The use of low-dose chest CT could reduce the risks associated with radiation exposure, and therefore potentially allow a more frequent application of CT to characterize the lungs and optimize mechanical ventilation in patients with ARDS.

## Thoracic-Computed Tomography Analysis in COVID-19 Patients

Thoracic-computed tomography is the most sensitive imaging technique for the diagnosis of COVID-19 lung involvement (Chung et al., 2020; Shi et al., 2020), showing diffuse infiltrates, especially in lungs periphery, ranging from ground-glass opacities to parenchymal consolidations. Multiple radiological patterns were observed at different times throughout the course of the disease. Lanza et al. (2020) analyzed 222 patients with RT-PCR positive for SARS-COV 2 virus who received a thoracic-computed tomography at admission because of dyspnea or desaturation. Compromised lung volume was the most accurate outcome predictor (logistic regression, *p* < 0.001). Tomographic lung volume loss in the range of 6–23% increased the risk for oxygenation support and ranges above 23% increased the risk for patient intubation. Compromised lung volume % showed a negative correlation with PaO2/FiO2 ratio (*p* < 0.001) and was a risk factor for inhospital mortality (*p* < 0.001).

The Fleischner Society published a consensus statement on the use of chest radiography and computed tomography during the COVID-19 pandemic to guide physicians in the use of chest imaging in the management of COVID-19 (Kwee and Kwee, 2020; Rubin et al., 2020). The statement noted that uncertainty still exists whether thoracic tomography should be used as a stand-alone screening tool, or as an adjunct tool to RT-PCR tests, to exclude occult infection before immunosuppression or surgery in regions with high prevalence of COVID-19 (Kwee and Kwee, 2020; Rubin et al., 2020).

The statement indicated thoracic tomography for patients with moderate to severe respiratory symptoms to demonstrate features of SARS-COV 2 infection or alternative diagnosis (Rubin et al., 2020). The statement also suggested guidance in reporting thoracic tomography findings are potentially attributable to COVID-19 pneumonia (Rubin et al., 2020) 1. typical appearance, 2. indeterminate appearance, 3. atypical appearance, and 4. negative for pneumonia. For a proper COVID-19 pneumonia diagnosis it is decisive to associate thoracic tomography findings with RT-PCR results, clinical manifestations, and epidemiological data (Kwee and Kwee, 2020).

A total of 1,431 symptomatic and at a high risk for COVID-19 patients, reported in a meta-analysis, revealed a thoracic CT-pooled sensitivity of 94.6% (95% CI: 91.9% and 96.4%) and a pooled specificity of 46.0% (95% CI: 31.9% and 60.7%) in the detection of COVID-19 (Adams et al., 2020). Another meta-analysis showed that 10.6% of symptomatic patients with positive RT-PCR for SARS-COV-2 infection had normal thoracic tomography (Raptis et al., 2020).

Patients with COVID-19 and respiratory failure have an increased rate of 11.8% of pulmonary embolism. COVID-19 patients diagnosed with thrombo-embolic complications have a more than 5-fold increase in mortality (Berger et al., 2020; Zhang et al., 2020). In COVID-19 patients with hypoxemia and relatively poor lung parenchymal alterations in thoracic tomography, high D-dimer, low (Sakr et al., 2020) plasmatic fibrinogen, signs of pulmonary hypertension, or signs of venous thrombosis (lower legs or intravenous devices) should be investigated with angio-thoracic tomography or dual-energy CT scan to the possible diagnosis of pulmonary embolism (McFadyen et al., 2020; Yu et al., 2020; Zhou et al., 2020).

## Dual-Energy CT Scan in COVID-19

Dual-energy tomography, also known as spectral CT or dual source CT, is a computed tomography technique that uses two separate x-ray photon energy spectra, allowing the interrogation of materials that have different attenuation properties at different energies, being used to reconstruct numerous images type, including iodine maps of lungs perfusion. A dual-energy CT scan has been recently studied to investigate ventilation–perfusion relationships in COVID-19 patients. Ball et al. (2021) analyzed pulmonary gas and blood distribution using a technique for quantitative analysis of dual-energy computed tomography in 35 COVID-patients needing non-invasive or invasive mechanical ventilation. Lung aeration loss (percentage of normally aerated lung tissue) and the extent of gas/blood volume mismatch (percentage of non-aerated, perfused lung tissue—shunt; aerated, non-perfused dead space; and non-aerated/non-perfused regions) were evaluated. Compared to patients requiring non-invasive ventilation, patients requiring invasive ventilation had both a lower percentage of normally aerated lung tissue [median (interquartile range) 33% (24–49%) vs. 63% (44–68%), *p* < 0.001]; and a larger extent of gas/blood volume mismatch [43% (30–49%) vs. 25% (14–28%), *p* = 0.001], due to higher shunt [23% (15–32%) vs. 5% (2–16%), *p* = 0.001] and non-aerated/non-perfused regions [5% (3–10%) vs. 1% (0–2%), *p* = 0.001]. The PaO2/FiO2 ratio correlated positively with normally aerated tissue (*ρ* = 0.730, *p* < 0.001). In patients with severe COVID-19 pneumonia, the need for invasive mechanical ventilation and the degree of hypoxemia were associated with loss of lungs aeration and the extent of gas/blood volume mismatch.

Aydin et al. (2021) studied 40 patients with positive RT-PCR to SARS-COV-2 with dual-energy thoracic tomography. All the patients had perfusion deficits at dual-CT images. Their mean perfusion deficit severity score was 8.45 ± 4.66 (min.-max, 1–19). In 24 of the 40 patients (60%), perfusion deficits and parenchymal lesions matched completely. In 15 of the 40 patients (37.5%), there was a partial match. The perfusion deficit severity score had a significantly positive correlation with D-dimer, reactive-C-protein levels, and thoracic tomography severity score (a score that is used in ARDS patients to assess severity and prognostication of patients) (Abbasi et al., 2020). The authors observed that perfusion deficits are seen not only in opacification areas but also in parenchyma of normal appearance.

Grillet et al. (2020) studied 85 patients with COVID-19 with dual-energy angio-tomography with iodine contrast and pulmonary iodine map. They observed that twenty-nine patients (34%) were diagnosed with pulmonary artery thrombosis, mainly segmental (83%). Semi-quantitative analysis revealed parenchymal ischemia in 68% of the 85 patients with no significant difference between the patients with or without pulmonary artery thrombosis (23 vs. 35, *p* = 0.144). Volume of ischemia was greater in patients with pulmonary artery thrombosis [29 (IQR, 8–100) vs. 8 (IQR, 0–45) cm^3^, *p* = 0.041]. Pulmonary perfusion evaluated by iodine maps shows extreme heterogeneity in COVID-19 patients and lower iodine levels in normal parenchyma. Pulmonary dual-energy angio-tomography revealed a significant number of pulmonary ischemic areas even in the absence of visible pulmonary arterial thrombosis, which may reflect micro-thrombosis associated with COVID-19 pneumonia.

Dual-energy angio-tomography studies in COVID-19 patients helped to understand the macro- and microvasculature involvement of the lungs in this disease. This vascular micro-thrombosis leads to severe alterations in the ventilation/perfusion ratio of the lungs and to high percentages of patients with hypoxemia and challenging to ventilate lungs (Abbasi et al., 2020; Grillet et al., 2020; Aydin et al., 2021; Ball et al., 2021).

## Alternative Diagnosis to COVID-19 and Complications Revealed by Thoracic-Computed Tomography

The finding of peripheral, bilateral, ground-glass opacities, predominantly in the lower lobes of the lungs is the typical finding of COVID-19 pneumonia, but can be the presentation features of other types of viral pneumonia, especially influenza (Yin et al., 2020). In the cases of typical findings of COVID pneumonia in the thoracic CT, but a negative RT-PCR for SARS-COV 2, a second RT-PCR for SARS-COV-2 must be made, and if negative, a molecular panel for respiratory virus must be asked for a correct diagnosis. Other alternative diagnosis includes: acute interstitial pneumonias, drug-induced lung diseases, alveolar hemorrhage, and ANCA-associated vasculitis.

In the cases of confirmed COVID-19 pneumonia, thoracic CT and angio-tomography is very helpful in diagnosing complications such as pulmonary embolism, acute respiratory distress syndrome, superimposed pneumonia, barotrauma (pneumomediastinum, pneumothorax, subcutaneous emphysema, pleural effusion, and pericardial effusions), organizing pneumonia, COVID-19 progression lung disease, secondary fungal infections, signs of pulmonary fibrosis, and mechanical ventilation-induced lung injury (barotrauma, lung cavitation, and cysts) (Kwee and Kwee, 2020).

COVID-19-associated pulmonary aspergillosis is reported in around 3. 3% of COVID-19 patients, with severe ARDS-receiving corticosteroids or tocilizumab (Machado et al., 2021). Diagnosis is established after a median of 15 days of invasive mechanical ventilation with bronchoalveolar lavage positive galactomannan or aspergillus positive culture. Thoracic tomography findings are compatible with pulmonary aspergilloma or invasive pulmonary aspergillosis (Machado et al., 2021).

## Severity CT Score and Prognostication of COVID-19 Patients

Abbasi et al. (Yin et al., 2020) retrospectively studied 262 hospitalized COVID-19 patients with a severity score that divided the lungs into three zones: upper, middle, and lower zones analyzing the degree of lung involvement for each zone: score of 0 (no involvement); 1. <25% involvement; 2. 25% to less than 50% involvement; 3. 50% to less than 75% involvement; and 4. ≥75% involvement. They observed a significant correlation between the CT severity score and rapidity of decline under the clinical condition of time to death, time to ICU admission, and time to intubation. Multivariate regression analysis showed increasing odds of inhospital death associated with a higher CT severity score at admission.

Popadic et al. (2021) studied 160 consecutive critically ill patients with the diagnosis of COVID-19 with moderate to severe ARDS observed in a multivariate analysis that the factors independently associated with mortality were IL-6, serum albumin, D-dimer, and thoracic-computed tomography score at admission.

Recently, Szabó et al. (2021) used artificial intelligence-based thoracic-computed tomography alterations quantification in patients with COVID-19 analyzed five lung regions upper right, middle right, lower right, upper left, and lower left lobes regarding lung volume and % of affected lung. They built a severity score that was calculated by a deep learning model based on the quantitative measurements. They observed that the artificial intelligence severity score was significantly associated with worse clinical outcomes. They concluded that their results provided personalized probabilities of adverse inhospital outcomes that might assist decision making in patients with COVID-19 lungs involvement that was not prospectively validated yet (Corrêa et al., 2021).

## Conclusion

In conclusion, thoracic tomography of the lungs in ARDS or COVID-19 patients can help to a better diagnosis of pulmonary involvement, classify its severity and make alternative diagnoses and detect possible complications. Thoracic-computed tomography can also help the intensivists adjust PEEP, tidal volume, and patient position during mechanical ventilation and follow-up of the ARDS patients during their hospital stay. Whether severity scores based on thoracic-computed tomography and artificial intelligence may help the clinical prognosis of patients with COVID-19 and ARDS remains to be determined.

## References

[B1] Abbasi B. Akhavan R. Ghamari Khameneh A. Zandi B. Farrokh D. Pezeshki Rad M. (2020). Evaluation of the Relationship between Inpatient COVID-19 Mortality and Chest CT Severity Score. Am. J. Emerg. Med. S0735-6757 (20), 30851–30852. 10.1016/j.ajem.2020.09.056 PMC7521211 33039235

[B2] Adams H. J. A. Kwee T. C. Yakar D. Hope M. D. Kwee R. M. (2020). Systematic Review and Meta-Analysis on the Value of Chest CT in the Diagnosis of Coronavirus Disease (COVID-19): Sol Scientiae, Illustra Nos. Am. J. Roentgenology 215, 1342–1350. 10.2214/AJR.20.23391 32478562

[B3] Amato M. B. P. Barbas C. S. V. Medeiros D. M. Magaldi R. B. Schettino G. P. Lorenzi-Filho G. (1998). Effect of a Protective-Ventilation Strategy on Mortality in the Acute Respiratory Distress Syndrome. N. Engl. J. Med. 338 (6), 347–354. 10.1056/nejm199802053380602 9449727

[B4] Aydin S. Kantarci M. Karavas E. Unver E. Yalcin S. Aydin F. (2021). Lung Perfusion Changes in COVID-19 Pneumonia: a Dual Energy Computerized Tomography Study. Bjr 94 (1125), 20201380. 10.1259/bjr.20201380 34415201 PMC9327758

[B5] Ball L. Robba C. Herrmann J. Gerard S. Xin Y. Mandelli M. (2021). Lung Distribution of Gas and Blood Volume in Critically Ill COVID-19 Patients: a Quantitative Dual-Energy Computed Tomography Study. Crit. Care 25, 214. 10.1186/s13054-021-03610-9 34154635 PMC8215486

[B6] Barbas C. S. Matos G. F. Amato M. B. Carvalho C. R. (2012). Goal-oriented Respiratory Management for Critically Ill Patients with Acute Respiratory Distress Syndrome. Crit. Care Res. Pract. 2012, 952168. 10.1155/2012/952168 22957224 PMC3432327

[B7] Barbas C. S. V. Hoelz C. Capellozzi V. L. (2003). Anesthesia, Pain, Intensive Care and Emergency Medicine. Gullo 117, 477–482. 10.1007/978-88-470-2215-7

[B8] Barbas C. S. V. de Matos G. F. J. (2019). “Recruitment Manoeuvres,” in ERS Practical Handbook. Invasive Mechanical Ventilation. Editors Heunks L. Schultz M. J.. Sheffield: European Respiratory Society, 185–194. 10.1183/9781849841221.030118

[B9] Barbas C. S. V. Ísola A. M. Caser E. B. (2014). What Is the Future of Acute Respiratory Distress Syndrome after the Berlin Definition? Curr. Opin. Crit. Care 20 (1), 10–16. 10.1097/mcc.0000000000000058 24316666

[B10] Barbas C. S. V. Nemer S. N. (2018). Lung Recruitment and Positive End-Expiratory Pressure Titration in Patients with Acute Respiratory Distress Syndrome. JAMA 319 (9), 933. 10.1001/jama.2017.21840 29509859

[B11] Berger J. S. Kunichoff D. Adhikari S. Ahuja T. Amoroso N. Aphinyanaphongs Y. (2020). Prevalence and Outcomes of D-Dimer Elevation in Hospitalized Patients with COVID-19. Atvb 40 (10), 2539–2547. 10.1161/atvbaha.120.314872 PMC7505147 32840379

[B12] Bombino M. Gattinoni L. Pesenti A. Pistolesi M. Miniati M. (1991). The Value of Portable Chest Roentgenography in Adult Respiratory Distress Syndrome. Chest 100 (3), 762–769. 10.1378/chest.100.3.762 1889270

[B13] Borges J. B. Okamoto V. N. Matos G. F. J. Caramez M. P. R. Arantes P. R. Barros F. (2006). Reversibility of Lung Collapse and Hypoxemia in Early Acute Respiratory Distress Syndrome. Am. J. Respir. Crit. Care Med. 174 (3), 268–278. 10.1164/rccm.200506-976oc 16690982

[B14] Caser E. B. Zandonade E. Pereira E. Gama A. M. C. Barbas C. S. V. (2014). Impact of Distinct Definitions of Acute Lung Injury on its Incidence and Outcomes in Brazilian ICUs. Crit. Care Med. 42 (3), 574–582. 10.1097/01.ccm.0000435676.68435.56 24158166

[B15] Cavalcanti A. B. Cavalcanti A. B. Suzumura É. A. Laranjeira L. N. Paisani D. M. Damiani L. P. Writing Group for the Alveolar Recruitment for Acute Respiratory Distress Syndrome Trial (ART) Investigators (2017). Effect of Lung Recruitment and Titrated Positive End-Expiratory Pressure (PEEP) vs Low PEEP on Mortality in Patients with Acute Respiratory Distress Syndrome: A Randomized Clinical Trial. JAMA 318 (14), 1335–1345. 10.1001/jama.2017.14171 28973363 PMC5710484

[B16] Chiumello D. Langer T. Vecchi V. Luoni S. Colombo A. Brioni M. (2014). Low-dose Chest Computed Tomography for Quantitative and Visual Anatomical Analysis in Patients with Acute Respiratory Distress Syndrome. Intensive Care Med. 40, 691–699. 10.1007/s00134-014-3264-1 24647812

[B17] Chung M. Bernheim A. Mei X. Zhang N. Huang M. Zeng X. (2020). CT Imaging Features of 2019 Novel Coronavirus (2019-nCoV). Radiology 295, 202–207. 10.1148/radiol.2020200230 32017661 PMC7194022

[B18] Corrêa T. D. Midega T. D. Timenetsky K. T. Cordioli R. L. Barbas C. S. Silva Júnior M. (2021). Clinical Characteristics and Outcomes of COVID-19 Patients Admitted to the Intensive Care Unit during the First Year of the Pandemic in Brazil: a Single center Retrospective Cohort Study. Einstein (São Paulo). 19, eAO6739. 10.31744/einstein_journal/2021AO6739 34878071 PMC8664289

[B19] Cressoni M. Chiumello D. Carlesso E. Chiurazzi C. Amini M. Brioni M. (2014). Compressive Forces and Computed Tomography-Derived Positive End-Expiratory Pressure in Acute Respiratory Distress Syndrome. Anesthesiology.121 (3), 572–581. 10.1097/aln.0000000000000373 25050573

[B20] de Matos G. F. Stanzani F. Passos R. H. Fontana M. F. Albaladejo R. Caserta R. E. (2012). How Large Is the Lung Recruitability in Early Acute Respiratory Distress Syndrome: a Prospective Case Series of Patients Monitored by Computed Tomography. Crit. Care 16, R4. 10.1186/cc10602 22226331 PMC3396229

[B21] Gattinoni L. Pelosi P. Crotti S. Valenza F. (1995). Effects of Positive End-Expiratory Pressure on Regional Distribution of Tidal Volume and Recruitment in Adult Respiratory Distress Syndrome. Am. J. Respir. Crit. Care Med. 151 (6), 1807–1814. 10.1164/ajrccm.151.6.7767524 7767524

[B22] Gattinoni L. Pesenti A. Carlesso E. (2013). Body Position Changes Redistribute Lung Computed-Tomographic Density in Patients with Acute Respiratory Failure: Impact and Clinical Fallout through the Following 20 Years. Intensive Care Med. 39 (11), 1909–1915. 10.1007/s00134-013-3066-x 24026295

[B23] Gattinoni L. Taccone P. Carlesso E. Marini J. J. (2013). Prone Position in Acute Respiratory Distress Syndrome. Rationale, Indications, and Limits. Am. J. Respir. Crit. Care Med. 188 (11), 1286–1293. 10.1164/rccm.201308-1532ci 24134414

[B24] Gattinoni L. Caironi P. Cressoni M. Chiumello D. Ranieri V. M. Quintel M. (2006). Lung Recruitment in Patients with the Acute Respiratory Distress Syndrome. N. Engl. J. Med. 354, 1775–1786. 10.1056/NEJMoa052052 16641394

[B25] Grillet F. Busse-Coté A. Calame P. Behr J. Delabrousse E. Aubry S. (2020). COVID-19 Pneumonia: Microvascular Disease Revealed on Pulmonary Dual-Energy Computed Tomography Angiography. Quant Imaging Med. Surg. 10 (9), 1852–1862. 10.21037/qims-20-708 32879862 PMC7417764

[B26] Guérin C. Reignier J. Richard J. C. Beuret P. Gacouin A. Boulain T. PROSEVA Study Group (2013). Prone Positioning in Severe Acute Respiratory Distress Syndrome. N. Engl. J. Med. 368 (23), 2159–2168. 10.1056/NEJMoa1214103 23688302

[B27] Kwee T. C. Kwee R. M. (2020). Chest CT in COVID-19: What the Radiologist Needs to Know. Radiographics 40 (7), 1848–1865. 10.1148/rg.2020200159 33095680 PMC7587296

[B28] Langer M. Mascheroni D. Marcolin R. Gattinoni L. (1988). The Prone Position in ARDS Patients. Chest 94 (1), 103–107. 10.1378/chest.94.1.103 3383620

[B29] Lanza E. Muglia R. Bolengo I. Santonocito O. G. Lisi C. Angelotti G. (2020). Quantitative Chest CT Analysis in COVID-19 to Predict the Need for Oxygenation Support and Intubation. Eur. Radiol. 30 (12), 6770–6778. 10.1007/s00330-020-07013-2 32591888 PMC7317888

[B30] Machado M. Valerio M. Álvarez‐Uría A. Olmedo M. Veintimilla C. Padilla B. COVID-19 Study Group (2021). Invasive Pulmonary Aspergillosis in the COVID‐19 Era: An Expected New Entity. Mycoses 64 (2), 132–143. 10.1111/myc.13213 33210776 PMC7753705

[B31] McFadyen J. D. Stevens H. Peter K. (2020). The Emerging Threat of (Micro)Thrombosis in COVID-19 and its Therapeutic Implications. Circ. Res. 127 (4), 571–587. 10.1161/circresaha.120.317447 32586214 PMC7386875

[B32] Pelosi P. Ball L. Barbas C. S. V. Bellomo R. Burns K. E. A. Einav S. (2021). Personalized Mechanical Ventilation in Acute Respiratory Distress Syndrome. Crit. Care 25 (1), 250. 10.1186/s13054-021-03686-3 34271958 PMC8284184

[B33] Pelosi P. D'Andrea L. Vitale G. Pesenti A. Gattinoni L. (1994). Vertical Gradient of Regional Lung Inflation in Adult Respiratory Distress Syndrome. Am. J. Respir. Crit. Care Med. 149 (1), 8–13. 10.1164/ajrccm.149.1.8111603 8111603

[B34] Pelosi P. D'Onofrio D. Chiumello D. Paolo S. Chiara G. Capelozzi V. L. (2003). Pulmonary and Extrapulmonary Acute Respiratory Distress Syndrome Are Different. Eur. Respir. J. 22:48s–56s. 10.1183/09031936.03.00420803 12946001

[B35] Pesenti A. Musch G. Lichtenstein D. Mojoli F. Amato M. B. P. Cinnella G. (2016). Imaging in Acute Respiratory Distress Syndrome. Intensive Care Med. 42, 686–698. 10.1007/s00134-016-4328-1 27033882

[B36] Popadic V. Klasnja S. Milic N. Rajovic N. Aleksic A. Milenkovic M. (2021). Predictors of Mortality in Critically Ill COVID-19 Patients Demanding High Oxygen Flow: A Thin Line between Inflammation, Cytokine Storm, and Coagulopathy. Oxid Med. Cel Longev 2021, 6648199. 10.1155/2021/6648199 PMC8081622 33968298

[B37] Ranieri V. M. Ranieri V. M. Rubenfeld G. D. Thompson B. T. Ferguson N. D. Caldwell E. ARDS Definition Task Force (2012). Acute Respiratory Distress Syndrome: the Berlin Definition. JAMA 307 (23), 2526–2533. 10.1001/jama.2012.5669 22797452

[B38] Raptis C. A. Hammer M. M. Short R. G. Shah A. Bhalla S. Bierhals A. J. (2020). Chest CT and Coronavirus Disease (COVID-19): a Critical Review of the Literature to Date. Am. J. Roentgenology 215, 839–842. 10.2214/AJR.20.23202 32298149

[B39] Rubin G. D. Ryerson C. J. Haramati L. B. Sverzellati N. Kanne J. P. Raoof S. (2020). The Role of Chest Imaging in Patient Management during the COVID-19 Pandemic. Chest 158 (1), 106–116. 10.1016/j.chest.2020.04.003 32275978 PMC7138384

[B40] Sakr Y. Giovini M. Leone M. Pizzilli G. Kortgen A. Bauer M. (2020). Pulmonary Embolism in Patients with Coronavirus Disease-2019 (COVID-19) Pneumonia: a Narrative Review. Ann. Intensive Care 10 (1), 124. 10.1186/s13613-020-00741-0 32953201 PMC7492788

[B41] Shi H. Han X. Jiang N. Cao Y. Alwalid O. Gu J. (2020). Radiological Findings from 81 Patients with COVID-19 Pneumonia in Wuhan, China: a Descriptive Study. Lancet Infect. Dis. 20, 425–434. 10.1016/s1473-3099(20)30086-4 32105637 PMC7159053

[B42] Simon M. Braune S. Laqmani A. Metschke M. Berliner C. Kalsow M. (2016). Value of Computed Tomography of the Chest in Subjects with ARDS: A Retrospective Observational Study. Respir. Care. 61 (3), 316–323. 10.4187/respcare.04308 26647453

[B43] Szabó I. V. Simon J. Nardocci C. Kardos A. S. Nagy N. Abdelrahman R. H. (2021). The Predictive Role of Artificial Intelligence-Based Chest CT Quantification in Patients with COVID-19 Pneumonia. Tomography 7 (4), 697–710. 10.3390/tomography7040058 34842822 PMC8628928

[B44] Yin Z. Kang Z. Yang D. Ding S. Luo H. Xiao E. (2020). A Comparison of Clinical and Chest CT Findings in Patients with Influenza A (H1N1) Virus Infection and Coronavirus Disease (COVID-19). Am. J. Roentgenology 215, 1065–1071. 10.2214/AJR.20.23214 32452731

[B45] Yu Y. Tu J. Lei B. Shu H. Zou X. Li R. (2020). Incidence and Risk Factors of Deep Vein Thrombosis in Hospitalized COVID-19 Patients. Clin. Appl. Thromb. Hemost. 26, 107602962095321. 10.1177/1076029620953217 PMC7457409 32854513

[B46] Zhang L. Yan X. Fan Q. Liu H. Liu X. Liu Z. (2020). D‐dimer Levels on Admission to Predict In‐hospital Mortality in Patients with Covid‐19. J. Thromb. Haemost. 18 (6), 1324–1329. 10.1111/jth.14859 32306492 PMC7264730

[B47] Zhou F. Yu T. Du R. Fan G. Liu Y. Liu Z. (2020). Clinical Course and Risk Factors for Mortality of Adult Inpatients with COVID-19 in Wuhan, China: a Retrospective Cohort Study. The Lancet 395, 1054–1062. 10.1016/s0140-6736(20)30566-3 PMC7270627 32171076

